# The plasma peptides of Alzheimer’s disease

**DOI:** 10.1186/s12014-021-09320-2

**Published:** 2021-06-28

**Authors:** Angelique Florentinus-Mefailoski, Peter Bowden, Philip Scheltens, Joep Killestein, Charlotte Teunissen, John G. Marshall

**Affiliations:** 1 Ryerson Analytical Biochemistry Laboratory (RABL), Department of Chemistry and Biology, Faculty of Science, Ryerson University, 350 Victoria St., Toronto, ON Canada; 2 Alzheimer Center, Dept of Neurology, Amsterdam University Medical Centers, Vrije Universiteit, Amsterdam Neuroscience, Amsterdam, The Netherlands; 3 MS Center, Dept of Neurology, Amsterdam University Medical Centers, Vrije Universiteit, Amsterdam Neuroscience, Amsterdam, The Netherlands; 4 Neurochemistry Lab and Biobank, Dept of Clinical Chemistry, Amsterdam University Medical Centers, Vrije Universiteit, Amsterdam Neuroscience, Amsterdam, The Netherlands; 5 International Biobank of Luxembourg (IBBL), Luxembourg Institute of Health (Formerly CRP Sante Luxembourg), Strassen, Luxembourg

**Keywords:** Alzheimer's, Plasma, Peptides, Peptidome, Mass spectrometry, Organic extraction, C18, Alzheimer’s dementia, Human EDTA plasma, Nano chromatography, Electrospray ionization tandem mass spectrometry, LC–ESI–MS/MS, Linear quadrupole ion trap, Discovery of variation, Random and independent sampling, Chi square test and ANOVA, SQL SERVER & R

## Abstract

**Background:**

A practical strategy to discover proteins specific to Alzheimer’s dementia (AD) may be to compare the plasma peptides and proteins from patients with dementia to normal controls and patients with neurological conditions like multiple sclerosis or other diseases. The aim was a proof of principle for a method to discover proteins and/or peptides of plasma that show greater observation frequency and/or precursor intensity in AD. The endogenous tryptic peptides of Alzheimer’s were compared to normals, multiple sclerosis, ovarian cancer, breast cancer, female normal, sepsis, ICU Control, heart attack, along with their institution-matched controls, and normal samples collected directly onto ice.

**Methods:**

Endogenous tryptic peptides were extracted from blinded, individual AD and control EDTA plasma samples in a step gradient of acetonitrile for random and independent sampling by LC–ESI–MS/MS with a set of robust and sensitive linear quadrupole ion traps. The MS/MS spectra were fit to fully tryptic peptides within proteins identified using the X!TANDEM algorithm. Observation frequency of the identified proteins was counted using SEQUEST algorithm. The proteins with apparently increased observation frequency in AD versus AD Control were revealed graphically and subsequently tested by Chi Square analysis. The proteins specific to AD plasma by Chi Square with FDR correction were analyzed by the STRING algorithm. The average protein or peptide log_10_ precursor intensity was compared across disease and control treatments by ANOVA in the R statistical system.

**Results:**

Peptides and/or phosphopeptides of common plasma proteins such as complement C2, C7, and C1QBP among others showed increased observation frequency by Chi Square and/or precursor intensity in AD. Cellular gene symbols with large Chi Square values (χ2 ≥ 25, p ≤ 0.001) from tryptic peptides included KIF12, DISC1, OR8B12, ZC3H12A, TNF, TBC1D8B, GALNT3, EME2, CD1B, BAG1, CPSF2, MMP15, DNAJC2, PHACTR4, OR8B3, GCK, EXOSC7, HMGA1 and NT5C3A among others. Similarly, increased frequency of tryptic phosphopeptides were observed from MOK, SMIM19, NXNL1, SLC24A2, Nbla10317, AHRR, C10orf90, MAEA, SRSF8, TBATA, TNIK, UBE2G1, PDE4C, PCGF2, KIR3DP1, TJP2, CPNE8, and NGF amongst others. STRING analysis showed an increase in cytoplasmic proteins and proteins associated with alternate splicing, exocytosis of luminal proteins, and proteins involved in the regulation of the cell cycle, mitochondrial functions or metabolism and apoptosis. Increases in mean precursor intensity of peptides from common plasma proteins such as DISC1, EXOSC5, UBE2G1, SMIM19, NXNL1, PANO, EIF4G1, KIR3DP1, MED25, MGRN1, OR8B3, MGC24039, POLR1A, SYTL4, RNF111, IREB2, ANKMY2, SGKL, SLC25A5, CHMP3 among others were associated with AD. Tryptic peptides from the highly conserved C-terminus of DISC1 within the sequence MPGGGPQGAPAAAGGGGVSHRAGSRDCLPPAACFR and ARQCGLDSR showed a higher frequency and highest intensity in AD compared to all other disease and controls.

**Conclusion:**

Proteins apparently expressed in the brain that were directly related to Alzheimer’s including Nerve Growth Factor (NFG), Sphingomyelin Phosphodiesterase, Disrupted in Schizophrenia 1 (DISC1), the cell death regulator retinitis pigmentosa (NXNl1) that governs the loss of nerve cells in the retina and the cell death regulator ZC3H12A showed much higher observation frequency in AD plasma vs the matched control. There was a striking agreement between the proteins known to be mutated or dis-regulated in the brains of AD patients with the proteins observed in the plasma of AD patients from endogenous peptides including NBN, BAG1, NOX1, PDCD5, SGK3, UBE2G1, SMPD3 neuronal proteins associated with synapse function such as KSYTL4, VTI1B and brain specific proteins such as TBATA.

**Supplementary Information:**

The online version contains supplementary material available at 10.1186/s12014-021-09320-2.

## Introduction

Studies of Alzheimer’s Dementia (AD) seem to show that proteins from the brain may be circulating in the blood [1]. AD may be associated with aberrant gene expression and RNA metabolism [2, 3], accompanied by cell death and clearance of cells from the brain [4]. There have been many proteomic studies to date focused on finding biomarkers for neurological diseases [5]. A peptide extraction using SDS-PAGE followed by electro elution onto MALDI chips identified Fibrinogen β chain FGA/B, AHSG and SERPING1 and biomarkers of AD [6]. A complex procedure for isolating exosomes from a large volume of starting plasma using size exclusion chromatography identified super-abundant blood proteins such as immunoglobulins, HLA-A or HLB-B, SERPINS and tetraspanins [7]. It was previously established that organic extraction was an effective method to pre-fractionate serum peptides [8]. Differential staining of 2 dimensional PAGE indicated differences in apolipoprotein isoforms between AD versus idiopathic normal pressure hydrocephalus patients [9]. Plasma protein profiling of mild cognitive impairment and Alzheimer’s disease using iTRAQ quantitative proteomics identified apolipoproteins including clusterin (APOJ), transferrin, and Inter-alpha-trypsin inhibitor (ITIH4) [10]. Synuclein is thought to be a marker of Parkinson’s disease and it has been studied in AD [11]. Protein arrays showed that ectodysplasin A2 receptor (EDA2R), Poliovirus receptor (PVR) and discoidin domain receptor family, member 1 (DDR1) were potential biomarkers of AD [12]. An array of nucleic acid aptamers was used to identify 44 proteins that apparently showed modest enrichments in AD but that showed no significant protein interactions, and APOE was the strongest correlate with amyloid burden [13]. A panel of apolipoprotein and acute phase or common response proteins was shown to discriminate between levels of amyloid burden [14]. Haptoglobin, serpin, Alpha-2-antiplasmin, and Antithrombin-III as well as Complement C4-A were increased in first-onset schizophrenia patients [15]. Alzheimer risk was associated with variation in the copy number of the Complement Receptor 1 thus increasing C3b/C4b binding sites [16]. The activation of the complement system by the myelin sheath [17] is consistent with elevated plasma levels of complement C4 that correlated with Multiple Sclerosis disease activity [18].

The neurological conditions Schizophrenia and multiple sclerosis have both been linked to the function of the complement system that prunes neural connections [18]. Plasma levels of Complement 4a protein are increased in Alzheimer’s disease [19]. X-Aptamers identified C4A and ApoB as potential markers for schizophrenia from blood [20]. Complement and microglia cells of the innate immune system mediate early synapse loss in a mouse model of Alzheimer’s dementia [21]. High levels of complement proteins were observed in astrocyte-derived exosomes of Alzheimer disease [22]. Complement protein levels in astrocyte-derived exosomes were abnormal in mild cognitive impairment [23]. Complement C4, gelsolin and the 14-3-3 Epsilon scaffold (YWHAE) were observed in both the brain and blood [1, 24]. Autoantibody profiling of glioma serum samples using arrays identified the 14-3-3 adaptor/scaffold YWHAH [25]. The presence of autoantibodies may indicate that the mechanism of AD has an autoimmune component [26]. Neuroprotective effects of regulatory T cells were observed in a Alzheimer’s disease model [27]. Increased levels of 14-3-3 gamma and epsilon proteins were observed in the brain of patients with Alzheimer’s disease and Down syndrome [24]. Myosin 1b is known to promote axon formation by regulating actin wave propagation and thus the dynamics of the growth cone [28]. The Genetic Loci ASTN2, DPP4 and MAST4 were associated with hippocampal volume [29]. A BECN1 mutation mediated autophagic sequestration of amyloid oligomers and improved cognition in Alzheimer’s disease [30]. Profiling the human hippocampal proteome at all pathologic stages of Alzheimer’s disease revealed an increase in cytoskeleton associated proteins RIMS1 and GRIK4 and synaptic proteins, such as BSN, LIN7A, DLG2, -3, and -4 [31]. Associations were observed between Hippocampal Morphometry and Neuropathologic Markers of Alzheimer’s disease [32]. BAG-1 is a protective factor that is increased in the brains of AD patients [33–35]. There is evidence that targeting TNF may prevent inflammatory damage in AD [36, 37]. The provision of nerve growth factor (NGF) has been suggested as a treatment for AD [38, 39]. Striated Muscle Enriched Protein Kinase (SPEG) and UBE2L3 that may be structurally and functionally related to Ubiquitin Conjugating Enzyme E2 G1 (UBE2G1) were differentially methylated genes associated with cognitive impairment [40] and circulating UBE2G1 transcripts may have potential as biomarkers [41]. Disrupted in Schizophrenia 1 (DISC1), TRAF2 and NCK Interacting Kinase (TNIK) have been identified from genomic sequence experiments as risk factor in AD [42, 43]. DISC1 is known to be expressed in neurological tissue and found in the blood and has been linked to mechanisms of Alzheimer’s pathology [44–47].

Analysis of Prokaryotic and Eukaryotic protein samples by LC–ESI–MS/MS indicates that observation frequency is a more informative parameter than peptide intensity for relative protein quantification by LC–ESI–MS/MS [48]. Random and independent sampling [49] is required for inference by classical statistical methods and has detected and confidently identified some ≥ 14,000 human protein Gene Symbols with more than 5 fully tryptic peptides by the stringent X!TANDEM algorithm from plasma [50]. In contrast, the use of a form of transect sampling has detected a few hundred proteins in serum or plasma [51–54].

Each clinical sample must be partitioned into multiple sub-fractions to achieve sensitivity [55, 56] for random and independent sampling by analytical C18 LC–ESI–MS/MS [55] that creates a large computational challenge. The MS/MS spectra from thousands of LC–ESI–MS/MS experiments from multiple clinical treatments and sites may be fit to peptides by the X!TANDEM and SEQUEST algorithms [57, 58]. The 32-bit computer lacked the power to identify and compare all the peptides of all the proteins of the many sub-factions from each patient in a large multisite clinical experiment [59]. The combination of step wise organic partition [55], random and independent sampling by nano electrospray LC–ESI–MS/MS [49], and 64-bit computation with SQL SERVER/R [60] permitted the sensitive detection of peptides and/or phosphopeptides. Thus, variation in the cleavage of parent protein chains and complexes from human plasma were compared between AD versus control alongside other diseases and normal plasma by the classical statistical approaches of the Chi Square test of observation frequency, STRING analysis of the identified proteins and univariate or two-way ANOVA of protein and peptide intensity [61–64]. There was striking agreement between the results of LC–ESI–MS/MS of the blood peptides with the known proteins and genes that participate in the pathology of Alzheimer’s dementia and the analysis revealed new neurological proteins specific to AD in circulation.

## Materials and methods

### Materials

Human EDTA plasma with no identifying information were received and analyzed under the Ryerson Ethical Review Board Protocol REB 2015-207: Treatment-blinded, Alzheimer’s dementia (AD), Multiple sclerosis (MS) and institution-matched normals were obtained from Amsterdam University Medical Centers, Vrije Universiteit Amsterdam; ICU-Sepsis versus ICU Control EDTA plasma were obtained from Clinical Evaluation Research Unit, Kingston General Hospital, Kingston Ontario Canada; Ovarian and breast cancer samples along with female only controls were obtained from the Ontario Tumor bank of the Ontario Institute of Cancer Research, Toronto Ontario; Heart attack (venous and arterial) versus pre-operative orthopedic surgery controls were obtained from St Joseph’s Hospital of McMaster University; In addition, EDTA plasma samples collected onto ice as a baseline degradation controls were obtained from IBBL Luxembourg [49, 65]. C18 zip tips were obtained from Millipore (Bedford, MA), C18 HPLC resin was from Agilent (Zorbax 300 SB-C18 5-micron). Solvents were obtained from Caledon Laboratories (Georgetown, Ontario, Canada). All other salts and reagents were obtained from Sigma-Aldrich-Fluka (St Louis, MO) except where indicated.

### Sample preparation

A total of 12 AD and 12 normal Control Human EDTA plasma samples (200 μl) were precipitated with 9 volumes of acetonitrile (90% ACN) [8], followed by extraction of the pellet using a step gradient to achieve selectivity across sub-fractions and thus greater sensitivity [55]. Disposable plastic 2 ml sample tubes and plastic pipette tips were used to handle samples. The acetonitrile suspension was separated with a centrifuge at 12,000 RCF for 5 min. The acetonitrile supernatant, that contains few peptides, was collected, transferred to a fresh sample tube and dried in a rotary lyophilizer. The organic precipitate (pellet) that contains a large total amount of endogenous polypeptides [8] was manually re-suspended using a step gradient of increasing water content to yield 10 fractions from those soluble in 90% ACN to 10% ACN, followed by 100% H_2_O and then 5% formic acid [55]. The extracts were clarified with a centrifuge at 12,000 RCF for 5 min. The extracted sample fractions were dried under vacuum in a rotary lyophyllizer and stored at − 80 ºC for subsequent analysis.

### Preparative C18 chromatography

Preparative C18 separation provided the best results for peptides and phosphopeptides analysis in a “blind” test [66]. Solid phase extraction with C18 for LC–ESI–MS/MS was performed as previously described [8, 56, 64, 67, 68]. The C18 chromatography resin (Zip Tip) was wet with 65% acetonitrile before equilibration in water with 5% formic acid. The plasma extract was dissolved in 200 μl of 5% formic acid in water. The resin was washed with at least five volumes of the same binding buffer. The resin was eluted with ≥ 3 column volumes of 65% acetonitrile (2 µL) in 5% formic acid. In order to avoid cross-contamination the preparative C18 resin was discarded after a single use.

### LC–ESI–MS/MS

In order to entirely prevent any possibility of cross contamination, a new disposable nano analytical HPLC column and nano emitter was fabricated for recording each patient sample-fraction set. The ion traps were cleaned and tested for sensitivity with angiontensin and glu fibrinogen prior to recordings. The new column was conditioned and quality controlled with a mixture of three non-human protein standards [61] using a digest of Bovine Cytochrome C, Yeast alcohol dehydrogenase (ADH) and Glycogen Phosphorylase B to confirm the sensitivity and mass accuracy of the system prior to each patient sample set. The statistical validity of the linear quadrupole ion trap for LC–ESI–MS/MS of human plasma [55] was in agreement with the results from the 3D Paul ion trap [61, 62, 69, 70]. The stepwise extractions were collected and desalted over C18 preparative micro columns, eluted in 2 µL of 65% ACN and 5% formic acid, diluted ten-fold with 5% formic acid in water and 5% ACN, and immediately loaded manually into a 20 μl metal sample loop before injecting onto the analytical column via a Rheodyne injector. Endogenous peptide samples were analyzed over a discontinuous gradient generated at a flow rate of ~ 10 micro litres per minute with an Agilent 1100 series capillary pump and split upstream of the injector during recording to about ~ 200 nl per minute. The separation was performed with a C18 (150 mm × 0.15 mm) fritted capillary column. The acetonitrile profile was started at 5%, ramped to 12% after 5 min and then increased to 65% over ~ 90 min, remained at 65% for 5 min, decreased to 50% for 15 min and then declined to a final proportion of 5% prior to injection of the next step fraction from the same patient. The nano HPLC effluent was analyzed by ESI ionization with detection by MS and fragmentation by MS/MS with a linear quadrupole ion trap [71]. The instrument was set to collect the precursors for up to 200 milli seconds prior to MS/MS fragmentation with up to four independent MS/MS fragmentations per precursor ion. Individual, independent samples from disease, normal and ice cold control were precipitated, fractionated over a step gradient and collected over C18 for manual injection. The level of replication in the LC–ESI–MS-MS experiments was typically between 9 to 26 independent patient plasma samples for each of the treatments with 12 AD and 12 AD control samples fractionated for analysis.

### Correlation analysis

Previous comparisons of high-resolution versus low-resolution analysis of peptides have shown that different mass spectrometric instruments show strong agreement on the high abundance proteins but that the Linear Quadrupole Ion Trap has the advantage of being more sensitive, economical, uses less power and gives off much less heat as well as having the smallest bench-top foot print [64, 72–75]. Thus, it is possible to use a battery of Linear Quadrupole Ion Traps to make multi site clinical trials using random and independent sampling followed by targeted quantification using the same simple, sensitive and robust instrument [49, 65]. Correlation analysis of ion trap data was performed using a goodness of fit test by X!TANDEM [57] and by cross-correlation using SEQUEST [76] on separate servers to match tandem mass spectra to peptide sequences from the Homo sapiens RefSeq, Ensembl, SwissProt, including hypothetical proteins XP or Genomic loci [60, 68, 72]. Setting the mass tolerance to a range including   heavy isotopes generates a greater number of strong correlations to the protein in the expected protein standard thus reducing the total error in proteomics [48, 77, 78]. The X!TANDEM default ion trap data settings of ± 3 m/z from precursor peptides considered from 300 to 2000 m/z with a tolerance of 0.5 Da error in the fragments were used [56, 57, 62, 69, 70, 78]. Peptides from proteins may show phosphorylation at serine, threonine, and tyrosine (STY) and it is common post-translational modification of proteins [79]. The best fit peptide of the MS/MS spectra to fully tryptic and/or phosphotryptic peptides at charge states of + 2 versus + 3 were accepted with additional acetylation, or oxidation of methionine and with possible loss of water or ammonia. The resulting accession numbers, actual and estimated masses, correlated peptide sequences, peptide intensity and MS/MS fragments to peptide fit scores, resulting protein sequences and other associated data were captured and assembled together in an SQL Server relational database [60].

### Data sampling, sorting, transformation and visualization

Endogenous peptides with precursors greater than 10,000 (E4) arbitrary counts were searched as fully tryptic peptides and/or phosphopeptides, the results were combined, and compared in SQL Server/R. The protein p-values and FDR q-values were computed from organic extraction or chromatography of blood fluid and the peptide-to-protein distribution of the precursor ions of greater than ~ 10,000 (E4) counts were compared to a null (i.e. known false positive) model of noise or computer generated random MS/MS spectra [49, 61, 62, 69, 70, 77]. Peptides may be identified from the fit of MS/MS spectra to peptide sequences by X!TANDEM [57] that permits the accurate estimate of the type I error rate (p-value) of protein identification that may be corrected by the method Benjamini and Hochberg [80] to yield the FDR (q-value) [49, 55, 77]. Random or noise MS/MS spectra distributions were used to control the type I error of experimental MS/MS spectra correlations to tryptic peptides: The peptide and protein observation counts (frequency) may be analyzed using classical statistic methods such as Chi Square analysis [69, 81]. Log_10_ transformation of precursor intensity yields a normal distributions that permits comparison of peptide and protein expression levels by ANOVA [62, 63]. The SQL Server system permits the direct interrogation of the related data by the open source R statistical system without proteomic-specific software packages. The use of SQL/R has permitted the detailed statistical analysis of randomly and independently sampled LC–ESI–MS/MS data from multiple hospitals in parallel that would be requisite for a multisite clinical trial [50, 81]. The linear quadrupole ion trap provided the precursor ion intensity values and the peptide fragment MS/MS spectra. The peptides and proteins were identified from MS/MS spectra by X!TANDEM and were counted by the SEQUEST algorithm. Redundant correlations to MS/MS at different charge states or to different sequences may be a source of type I error that may be filtered out by a complex key in SQL Server. The MS and MS/MS spectra together with the results of the X!TANDEM and SEQUEST algorithms were parsed into an SQL Server database and filtered [60] before statistical and graphical analysis with the generic R data system [60–63, 72]. The peptide-to-protein correlation frequency counts for each gene symbol were summed over AD versus the matched control to correct the observation frequency for the Chi Square test using Eq. 1:

1 $${\text{(AD}}{ - }{\text{AD}}_{\_} {\text{control)}}^{2} {\text{/(AD}}_{\_} {\text{control + 1)}}$$

The precursor intensity data for MS/MS spectra were log_10_ transformed, tested for normality and analyzed across institution/study and diseases versus controls by means, standard errors and ANOVA [61–63]. The entirely independent analysis of the precursor intensity by ANOVA versus multiple treatments and controls was achieved using a 64-bit R server.

## Results

Partition of plasma samples using differential solubility in organic/water mixtures was combined with random and independent sampling by LC–ESI–MS/MS  and detected peptides from proteins that were more frequently observed and/or showed greater intensity in AD versus AD_control. Here four independent lines of evidence, Chi Square analysis of observation frequency, previously established structural/functional relationships from STRING, ANOVA analysis of peptide intensity, and agreement with the previous genetic or biochemical experiments, all indicated that there was significant statistical and biological variation in the peptides of AD patients compared to AD control and other diseases or normal plasma samples.

### LC–ESI–MS/MS

The pool of endogenous tryptic (TRYP) and/or tryptic phosphopeptides (STYP) were randomly and independently sampled by liquid chromatography, nano electrospray ionization and tandem mass spectrometry (LC–ESI–MS/MS) [49] from AD vs AD Control or other disease and normal plasma, and ice cold controls to serve as a baseline [65, 82]. Some 15,968,550 MS/MS spectra ≥ E4 intensity counts were correlated by the SEQUEST and X!TANDEM algorithms to match the MS/MS spectra to tryptic peptides within proteins. The correlations from SEQUEST were filtered to retain only the best fit by charge state and peptide sequence in SQL Server to avoid re-use of the same MS/MS spectra. The distinct results were then analyzed by the generic R statistical system in a matrix of disease and controls that reveals the set of blood peptides and proteins specific to each disease state. The statistical validity of the extraction and sampling system were previously established by computation of protein gene symbols p-values and FDR corrected q-values by the method of Benjamini and Hochberg [80] and frequency comparison to false positive noise or random MS/MS spectra [48, 61, 63, 69, 70, 77, 78].

### Frequency correction

**Fig. 1:**
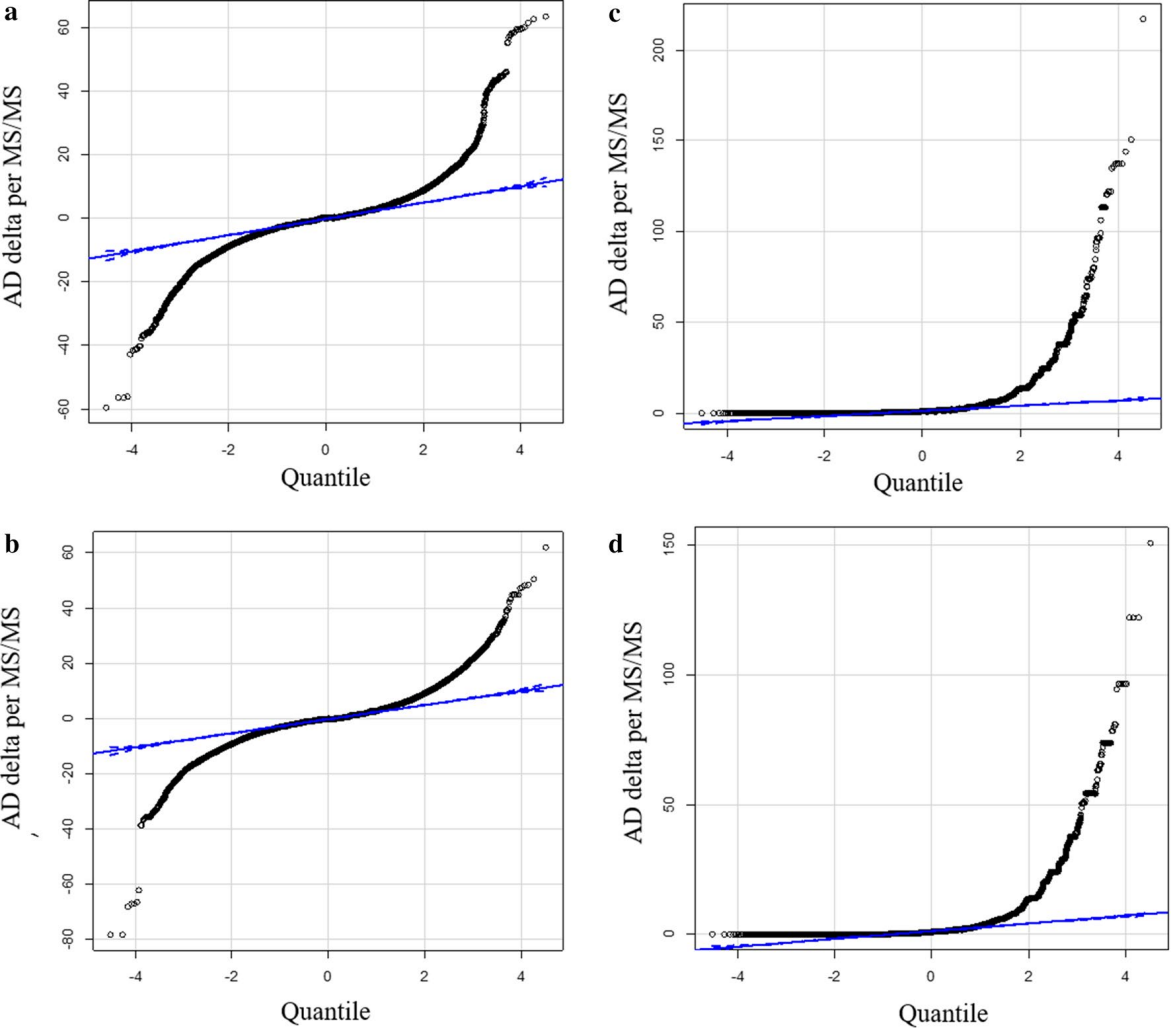
Quantile plots of the corrected difference in observation frequency and Chi Square values of the AD (n ≥ 10) versus Normal Control (n ≥ 9). Panels: **a** Quantile plot of the observation frequency of tryptic peptides from AD versus Normal Control.; **b** χ2 plot of the observation frequency of tryptic peptides from AD versus Normal Control tryptic peptides; **c** Quantile plot of the observation frequency of tryptic STYP peptides from AD versus AD control; **d** χ2 plot of the observation frequency of tryptic STYP peptides from AD versus AD control tryptic peptides

Chi Square (χ2) may be used to compare discrete, “counting” variables such as observation frequency. A total of 486,367 MS/MS ≥ E4 counts were collected from AD and 424,591 MS/MS ≥ E4 counts were collected from the AD Normal control plasma and these sums were used to correct observation frequency. Similar results were obtained from comparisons corrected on the basis of total correlation sum in each treatment (not shown). Removing the treatment-blind revealed many proteins that showed large increases or decreases in observation frequency between AD versus the matched AD normal resulting in large Chi Square values (Fig. 1).

### Comparison of AD to matched control by Chi square analysis

The gene symbols with the most significant Chi Square values (χ2 ≥ 90) from tryptic peptides included kinesin KIF12, disrupted in schizophrenia 1 (DISC1), the auto immune target olfactory receptor 8 (OR8B12) [83] and Monocyte Chemotactic Induced Zinc Finger CCCH Domain-Containing Protein Endoribonuclease (ZC3H12A) that regulates cell death [84]. Many proteins similar to olfactory receptors were observed with multiple peptide correlation by X!TANDEM and SEQUEST (Additional file 6: Table S6). The observation of many peptides correlated to proteins similar to olfactory receptors by both X!TANDEM and SEQUEST seem to indicate that genes for olfactory receptors are transcribed into RNA and give rise to  measurable amounts of translated proteins like other protein genes [85]. The most significant gene symbols (χ2 ≥ 90) from phosphotryptic peptides included MOK protein kinase, the truncated form of thioredoxin, Retinosa pigmentosa, the cell death regulator required for cone viability (NXNL1) and unknown small membrane protein 19 (SMIM19). A set of ~ 50 gene symbols showed a substantial difference ≥ 9 counts and a χ2 ≥ 25 (p ≤ 0.001) between AD versus the matched AD Control. For χ2 analysis the tryptic peptides (TRYP) were computed independently from the phospho-tryptic peptides. Common plasma proteins such as C2, C7, and C1QBP were observed to show significant differences in observation frequency between AD versus AD control. Specific peptides and/or phosphopeptides from cellular proteins, membrane proteins, nucleic acid binding proteins, signaling factors, metabolic enzymes and others including uncharacterized proteins showed significantly greater observation frequency in AD (Table 1). Gene symbols specific to AD included TNF, TBC1D8B, GALNT3, EME2, CD1B, BAG1, CPSF2, MMP15, DNAJC2, PHACTR4, OR8B3, GCK, EXOSC7, HMGA1 and NT5C3A among others from tryptic peptides. Similarly, increased frequency of tryptic phosphopeptides were observed from SLC24A2, CUX1, AHRR, C10orf90, MAEA, SRSF8, TBATA, TNIK, UBE2G1, PDE4C, PCGF2, KIR3DP1, TJP2, CPNE8, and NGF amongst others. The observation frequency of peptides from DISC1 was higher in AD compared to any other disease or control treatment. Observation frequency may be the best measure of relative abundance [48] and the full list of Chi Square results (χ2 ≥ 9, p ≤ 0.01) are found in the Supplemental Data that is the most important result of this study (Additional file 1: Table S1).

**Table 1:** AD specific proteins detected by fully tryptic peptides (TRYP)  and/or fully tryptic phosphopeptides (STYP) that show a Chi Square (χ2) value of ≥ 43.  The average Chi Square (χ2) value per gene symbol was computed in R

Gene_symbol	TRYP_X2	Proteins	Gene_symbol	STYP_X2	Proteins
KIF12	170	2	MOK	151	1
DISC1	104	29	SMIM19	96	2
OR8B12	96	1	NXNL1	94	1
ZC3H12A	94	1	SLC24A2	85	2
TNF	81	3	CUX1	78	1
TBC1D8B	74	1	AHRR	74	1
GALNT3	74	1	C10orf90	74	1
EME2	74	1	MAEA	74	1
CD1B	74	1	SRSF8	74	1
BAG1	73	2	TBATA	74	1
CPSF2	68	2	TNIK	74	1
MMP15	65	1	UBE2G1	74	2
DNAJC2	65	3	PDE4C	70	10
PHACTR4	64	1	PCGF2	69	2
OR8B3	64	1	KIR3DP1	65	1
GCK	64	1	TJP2	65	1
EXOSC7	64	1	CPNE8	63	2
HMGA1	63	4	NGF	59	1
NT5C3A	63	2	ZNF671	57	2
POLR1A	59	1	ADAMTS12	54	1
NET1	57	1	APC	54	1
MGC24039	57	1	ASAH2B	54	1
SYTL4	56	2	BRCA1	54	1
CLDN7	56	2	C11orf89	54	1
OSBPL1A	55	2	CBWD7	54	1
VTI1B	54	1	DAPK3	54	1
TRIM14	54	1	DCAF15	54	1
TPH1	54	1	GPR98	54	1
TNFRSF14	54	1	GRID2	54	1
SORBS3	54	1	HSP90AA2	54	1
SLC30A1	54	1	KIAA1467	54	1
RSRC2	54	1	KIR222	54	1
PDCD5	54	1	L3MBTL2	54	1
PANO	54	1	LOC389605	54	1
METTL17	54	1	NBS1	54	1
MATK	54	1	NPR2	54	3
ILK	54	1	OR8H2	54	1
FLJ00366	54	1	PDZD9	54	1
EPHX3	54	1	PTTG2	54	2
DYM	54	1	RAD52	54	1
CSNK2B-LY6G5B-1181	54	1	RALGAPA2	54	1
SPEG	54	4	SIRPA	54	3
CDH13	52	5	SLMAP	54	1
SLC36A2	51	5	SRM	54	1
SMPD3	51	3	TSPY8	54	1
WDR82	51	1	XP32	54	1
TRAF4	51	1	XPC	54	1
SGK3	51	1	RP11-632C17__A.1-001	54	1
NCAPD3	51	1	DENND3	51	2
GRXCR1	51	1	CDC27	51	1
ANKS6	49	1	FLJ32063	51	1
CYP4A11	48	8	LOC100129307	51	1
EXOC3L4	46	2	NOX1	51	1
POC1A	46	2	NUP210	51	1
FUT9	46	2	OGFOD3	51	1
MAP3K19	46	4	MST1R	50	1
CHD9	46	1	NEUROG2	50	1
USP30	44	1	PTPLA	50	2
DCUN1D2	44	1	PRKCD	49	1
BOK	44	1	MROH9	49	1
TAGLN2	44	2	CHMP3	48	3
TEX101	43	3	C2	48	9

The full set of gene symbols with Chi Square (χ2) ≥ is shown in Additional file 1: Table S1

**Table 2:** The STRING analysis of the AD specific protein network where corrected difference in observation frequency and the Chi Square value were both greater than 15 that showed: nodes, 1248; number of edges, 5604; average node degree, 8.98; avg. local clustering coefficient, 0.321; expected number of edges, 5362; PPI enrichment p-value, 0.000516

Term ID	Term description	Observed gene count	Background gene count	False discovery rate
KW-0025	Alternative splicing	821	10223	1.98E-18
KW-0963	Cytoplasm	379	4972	0.0178
GO:0005829	cytosol	405	4958	0.0000291
GO:0044444	cytoplasmic part	695	9377	0.0000291
GO:0005737	Cytoplasm	809	11238	0.0000436
GO:0005622	Intracellular	983	14286	0.0007
GO:0044424	Intracellular part	966	13996	0.0007
GO:0043226	Organelle	866	12432	0.0022
GO:0005623	Cell	1092	16271	0.0029
GO:0044464	Cell part	1090	16244	0.003
GO:0043229	Intracellular organelle	848	12193	0.0034
GO:0043231	Intracellular membrane-bounded organelle	729	10365	0.0096
GO:0044422	Organelle part	649	9111	0.0096
GO:0005768	Endosome	85	876	0.0158
GO:0043227	Membrane-bounded organelle	781	11244	0.0158
GO:0098576	Lumenal side of membrane	9	29	0.0219
GO:0055037	Recycling endosome	24	164	0.024
GO:0044446	Intracellular organelle part	627	8882	0.0287
GO:0071556	Integral component of lumenal side of endoplasmic reticulum	8	26	0.0404
GO:0003824	Catalytic activity	435	5592	0.0028
GO:0005488	Binding	830	11878	0.0174
GO:0008152	Metabolic process	696	9569	0.0108
GO:0051726	Regulation of cell cycle	115	1129	0.0108
GO:0010564	Regulation of cell cycle process	76	684	0.0175
GO:1901990	Regulation of mitotic cell cycle phase transition	47	351	0.0175
GO:0045786	Negative regulation of cell cycle	61	517	0.0186
GO:0010821	Regulation of mitochondrion organization	26	148	0.0187
GO:0044237	Cellular metabolic process	637	8797	0.0187
GO:0044238	Primary metabolic process	638	8808	0.0187
GO:0071704	Organic substance metabolic process	657	9135	0.0187
GO:1901987	Regulation of cell cycle phase transition	49	385	0.0187
GO:0008104	Protein localization	172	1966	0.0208
GO:0007346	Regulation of mitotic cell cycle	67	608	0.0212
GO:0006807	Nitrogen compound metabolic process	603	8349	0.0286
GO:0009056	Catabolic process	162	1859	0.0286
GO:0009987	Cellular process	994	14652	0.0286
GO:0010823	Negative regulation of mitochondrion organization	13	50	0.0286
GO:0044248	Cellular catabolic process	147	1646	0.0286
GO:0048583	Regulation of response to stimulus	306	3882	0.0286
GO:0051641	Cellular localization	186	2180	0.0286
GO:0080134	Regulation of response to stress	120	1299	0.0286
GO:0097190	Apoptotic signaling pathway	39	295	0.0286
GO:1901564	Organonitrogen compound metabolic process	401	5281	0.0286
GO:0032270	Positive regulation of cellular protein metabolic process	134	1496	0.0315
GO:0019538	Protein metabolic process	324	4194	0.0389
GO:0044403	Symbiont process	68	650	0.0389
GO:0007093	Mitotic cell cycle checkpoint	24	153	0.0434
GO:0044260	Cellular macromolecule metabolic process	472	6413	0.0434
GO:0016032	Viral process	61	571	0.0439
GO:0016197	Endosomal transport	27	185	0.0452
GO:0051128	Regulation of cellular component organization	191	2306	0.0452
GO:0051247	Positive regulation of protein metabolic process	139	1587	0.0452
GO:0043170	Macromolecule metabolic process	539	7453	0.0463

Keywords, GO components, processes and functions from STRING analysis of gene symbols with a χ2 ≥ 25 for AD versus AD control

The supporting information from the STRING analysis may be found in Additional files 2, 3, 4: Table S2–S4

### STRING network analysis

**Fig. 2:**
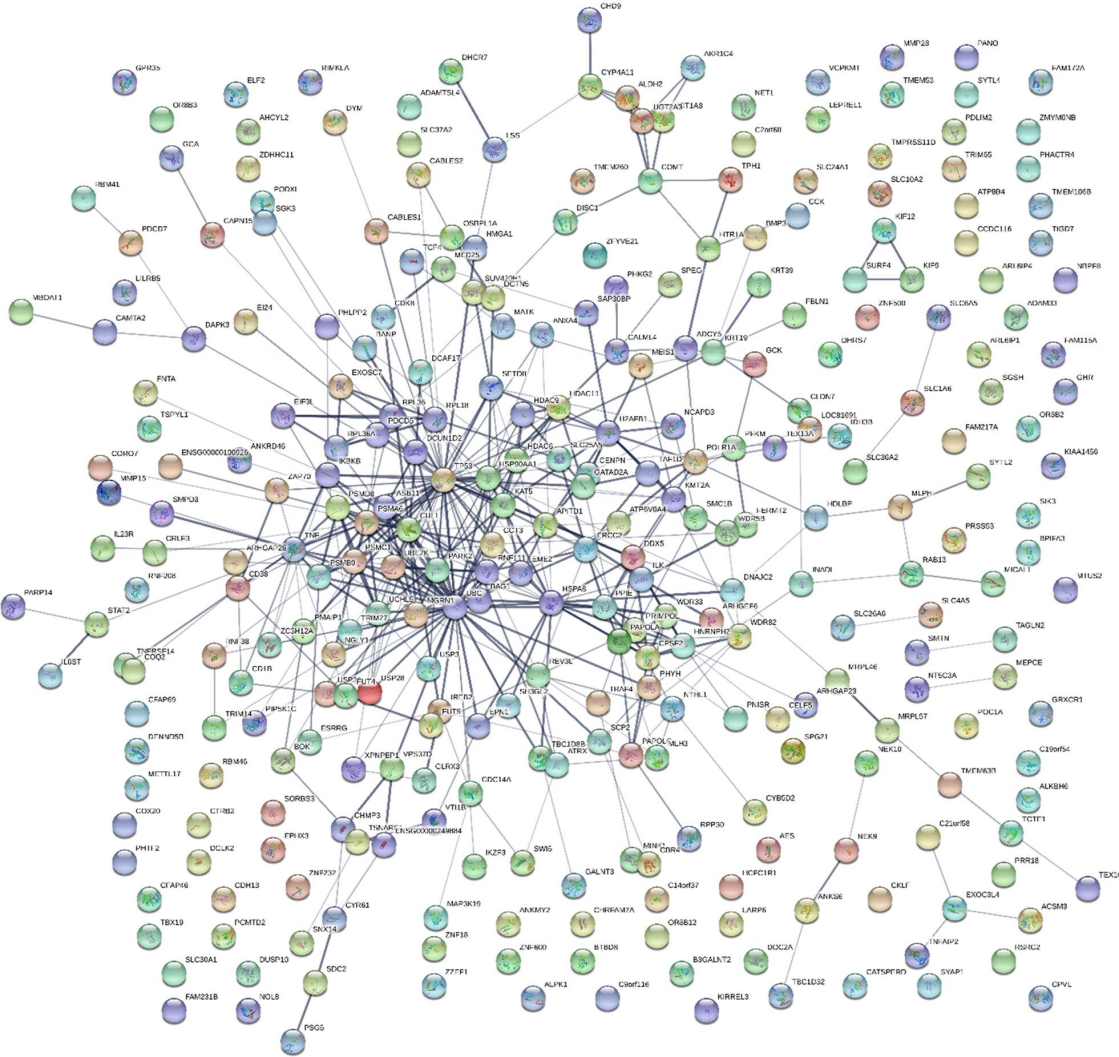
The AD STRING network where tryptic (TRYP) peptide frequency difference greater than 10 and Chi Square χ2 ≥ 25 (p < 0.001)

**Fig. 3:**
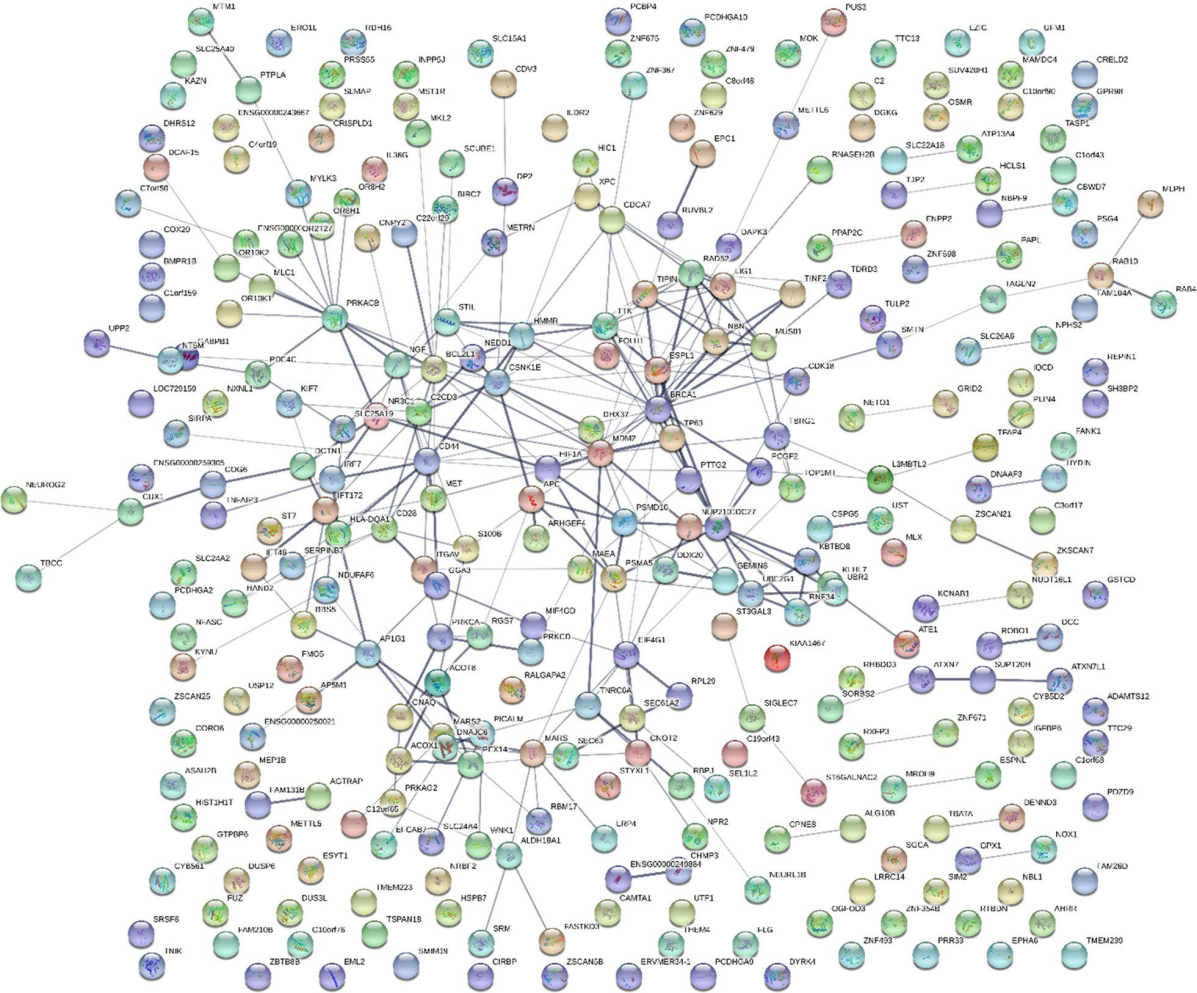
The AD STRING network where phospho-tryptic (STYP) and/or tryptic peptide frequency difference greater than 10 and Chi Square χ2 ≥ 25 (p < 0.001)

The gene symbols that varied between AD versus AD control with average Chi Square χ2 ≥ 25 (p ≤ 0.01) revealed a complex network of protein gene symbols [86] of 1163 nodes with 5017 edges (PPI enrichment p-value of 0.00602). Similarly, phosphotryptic peptides (STYP) with gene symbol Chi Square (χ2) ≥ 25 revealed a network of proteins [86] with 1224 nodes and 5066 edges (PPI enrichment p-value of 0.00342). For the purposes of illustration, the proteins that showed at least 9 greater observations (Delta) and χ2 values greater than 25 (p < 0.001) are shown as separate as tryptic (TRYP), versus phospho-tryptic (STYP), STRING networks (Figs. 2 and 3). STRING analysis showed an increase in cytoplasmic proteins and proteins associated with alternate splicing, exocytosis of luminal proteins, and proteins involved in the regulation of the cell cycle, mitochondrial functions or metabolism and apoptosis (Table 2). The full list of Gene Symbols from tryptic peptides, phospho tryptic peptides and the resulting STRING analysis may be found in Additional files 2, 3, 4, 5: Table S2−S5.

### Quantile box plots and ANOVA analysis across disease and control treatments

**Fig. 4:**
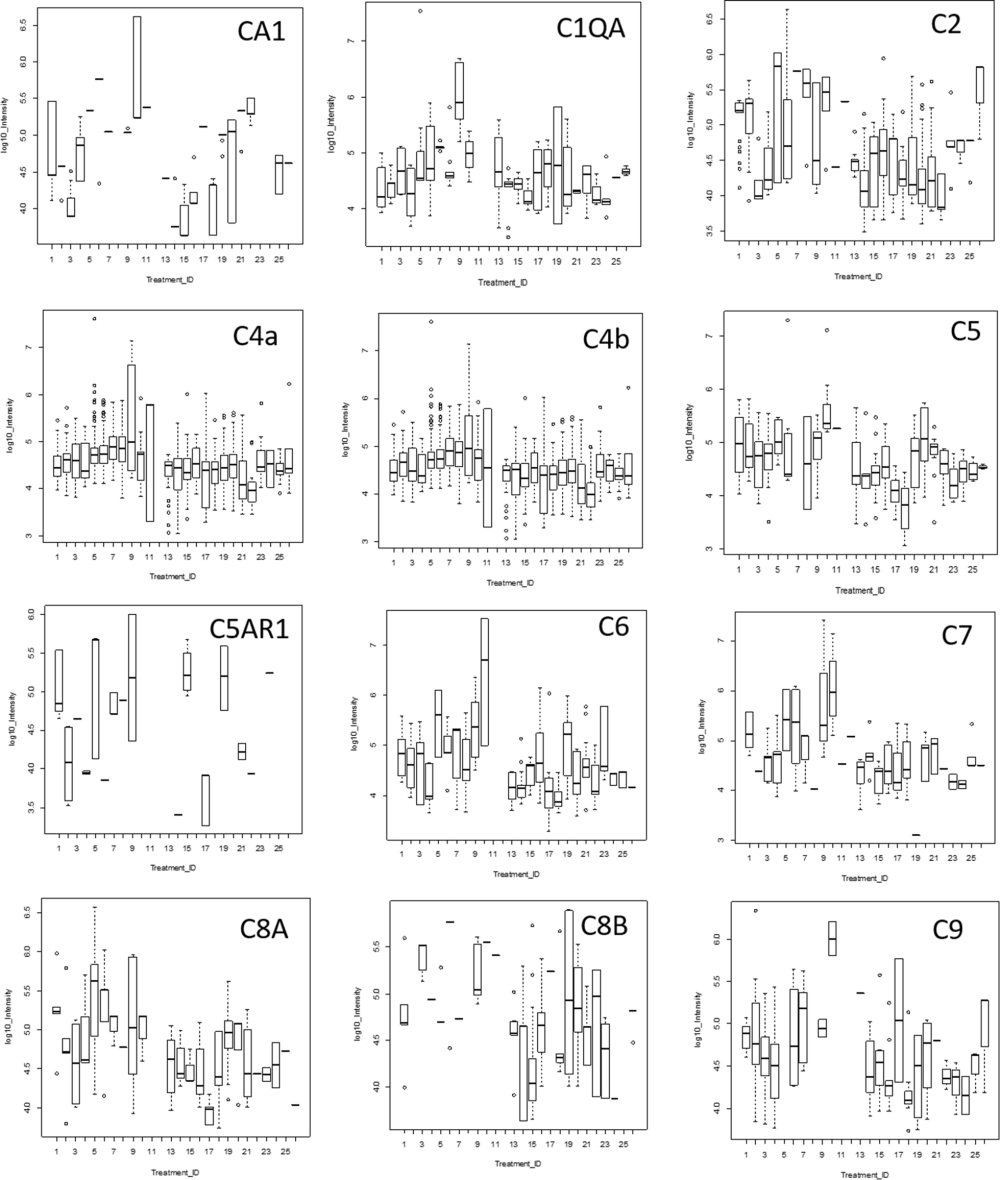
The distributions of log_10_ precursor intensity by quantile and box plots of complement proteins across the disease and control treatments. Treatment ID numbers: 1, Alzheimer normal; 2, Alzheimer’s normal control STYP; 3, Alzheimer’s dementia; 4, Alzheimer’s dementia STYP; 5, Cancer breast; 6, Cancer breast STYP; 7, Cancer control; 8, Cancer control STYP; 9, Cancer ovarian; 10, Cancer ovarian STYP; 11, Ice Cold; 12, Ice Cold STYP; 13, Heart attack Arterial; 14 Heart attack Arterial STYP; 15, Heart attack normal control, 16, Heart attack normal Control STYP; 17, Heart attack; 18, Heart attack STYP; 19, Multiple Sclerosis normal control; 20, Multiple sclerosis normal control STYP; 21, Multiple sclerosis; 22, Multiple Sclerosis STYP, 23 Sepsis; 24, Sepsis STYP; 25, Sepsis normal control; 26, Sepsis normal control STYP. There was significant effects of treatments and peptides by two-way ANOVA. Analysis of the proteins shown across treatments produced a significant F Statistic by one-way ANOVA. Note the C3 results were previously published [165]

**Fig. 5:**
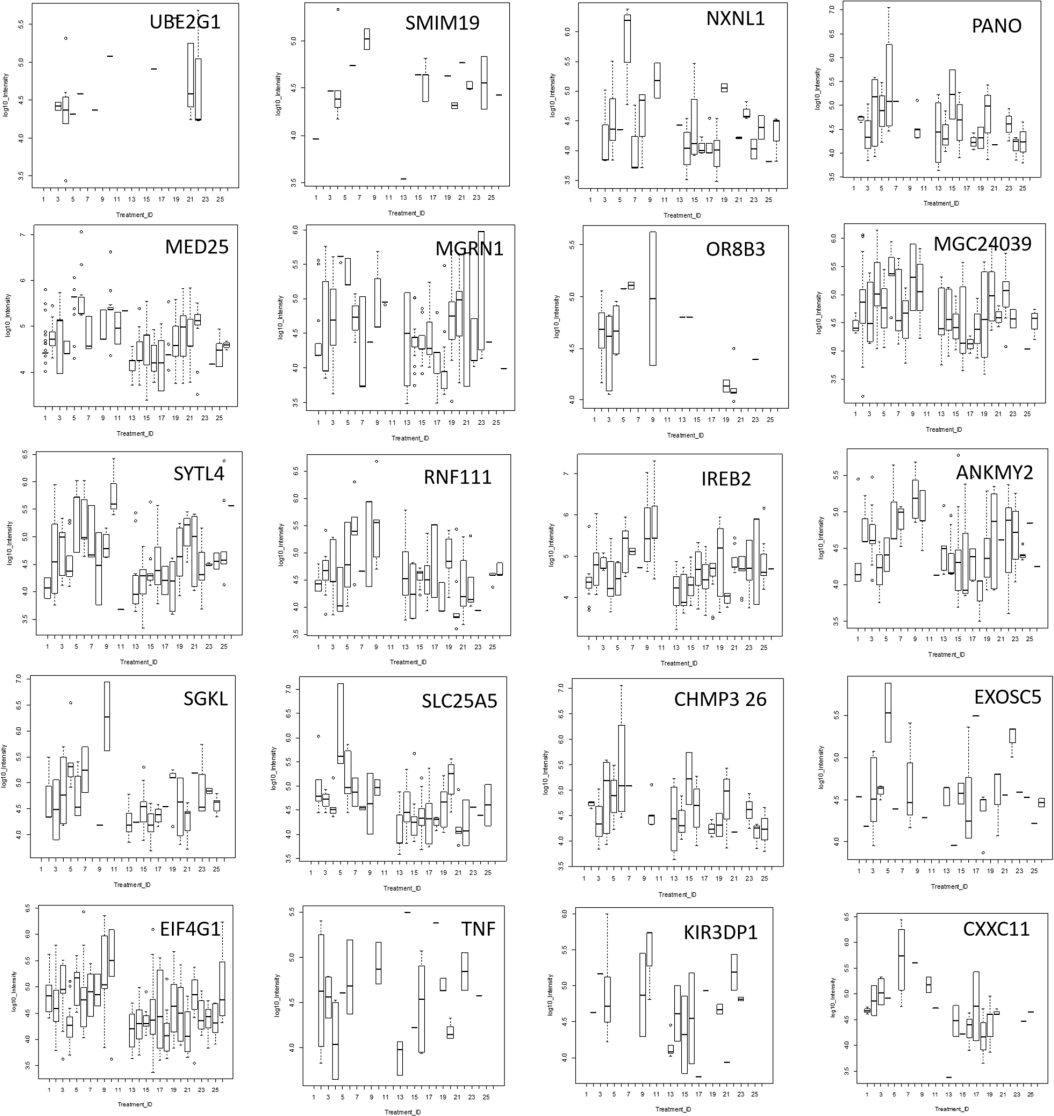
The distributions of log_10_precursor intensity by box plots of the cellular proteins across the disease and control treatments. Treatment ID numbers: 1, Alzheimer normal; 2, Alzheimer’s normal control STYP; 3, Alzheimer’s dementia; 4, Alzheimer’s dementia STYP; 5, Cancer breast; 6, Cancer breast STYP; 7, Cancer control; 8, Cancer control STYP; 9, Cancer ovarian; 10, Cancer ovarian STYP; 11, Ice Cold; 12, Ice Cold STYP; 13, Heart attack Arterial; 14 Heart attack Arterial STYP; 15, Heart attack normal control, 16, Heart attack normal Control STYP; 17, Heart attack; 18, Heart attack STYP; 19, Multiple Sclerosis normal control; 20, Multiple sclerosis normal control STYP; 21, Multiple Sclerosis; 22, Multiple sclerosis STYP, 23 Sepsis; 24, Sepsis STYP; 25, Sepsis normal control; 26, Sepsis normal control STYP. There was significant effects of treatments and peptides by two-way ANOVA. Analysis of the proteins shown across treatments produced a significant F Statistic by one-way ANOVA

ANOVA of peptide intensity is confounded by the different peptides observed within each protein [61] but may be an independent method to confirm the potential utility of peptides from gene symbols that showed increased observation frequency by Chi Square. Some proteins that showed greater observation frequency in AD also showed significant variation in protein or peptide precursor intensity compared to the AD controls and/or other disease and normal EDTA plasma by quantile box plots and ANOVA comparison. The mean precursor intensity values from gene symbols that varied by Chi Square from tryptic and/or phosphotryptic were subsequently analyzed by ANOVA [61] in R to look for proteins that showed significant variation in precursor intensity values across treatments [63, 64]. Common plasma complement proteins including C2, C7, and C1QBP were analyzed for variation in average peptide log_10_ intensity across treatments using ANOVA and box plots (Fig. 4). Analysis of the proteins with increased observation frequency in AD by precursor intensity using quantile box plots and/or ANOVA confirmed significant variation in cellular proteins UBE2G1, SMIM19, NXNL1, PANO, MED25, MGRN1, OR8B3, MGC24039, SYTL4, RNF111, IREB2, ANKMY2, SGKL, SLC25A5, CHMP3 26, EXOSC5 among others across disease treatments (Fig. 5).

### Agreement of AD specific proteins from plasma with previous biochemical and genetic data

There as striking agreement between the proteins observed to be specific to AD by LC–ESI–MS/MS and the proteins previously associated with AD by independent biochemical, genetic or genomic methods (Table 3).

**Table 3:** Agreement of AD specific LC–ESI–MS/MS results with previous genetic, genomic and biochemical experiments from an automated search of NCBI PubMed

Gene symbol	Protein name	Protein function	References
APC	Anaphase-promoting complex	The anaphase-promoting complex (APC) pathway was shown to regulate dendritic memory	[132]
APOE	Lipid binding and transport	Carboxyl-terminal-truncated Apolipoprotein E4 causes Neurodegeneration in mice	[94]
BAG1	BCL2 Associated Athanogene	Is associated with memory deficit in Alzheimer’s	[33, 34]
BRCA1	RING-Type E3 Ubiquitin Transferase	May have a role in neuron death in Alzheimer’s	[133]
C1Q & C9	Complement activation	Upregulation complement C1Q and C9 in AD	[134]
CDC27	APC component catalyzes the formation of cyclin B-ubiquitin conjugate	Knockdown of CDC27 leads to enhanced neurite growth	[135]
CDH13	calcium-dependent cell adhesion protein cadherin 13	Was implicated in Alzheimer’s	[136]
CHD9	Chromodomain Helicase DNA Binding Protein 9	Is closely related to a brain specific DNA remodelling enzyme	[137]
CUX CUX1	Cut Like Homeobox	Repressor of dendrite morphology and arborisation	[138] [138]
DAPK	The cell death-associated protein kinase family	May be therapeutic targets in AD	[98]
DISC1	Disrupted in Schizophrenia 1	Coiled-coil and leucine zippers that may mediate protein–protein interactions	[44, 46, 87, 126, 130]
EXOC3L4	Variants in the splicing regulatory elements?	Were associated with Alzheimer’s disease	[99]
FUT9	Fucosyltransferase IX	Amyloid β-peptide 1–42 modulates the expression activity	[139]
GALNT7	Acetylgalactosaminyltransferase 7, related to GALNT3	Genomic sequence data has related, with Alzheimer’s disease	[140]
GRID2	Glutamate Ionotropic Receptor Delta Type Subunit 2	Is important for the function of the NMDA receptor that is a therapeutic target in AD	[100]
GRXCR1	Glutaredoxin And Cysteine Rich Domain Containing 1	Was observed in the plasma of AD patients and Glutaredoxin was released to the cerebrospinal fluid in the early stages of AD	[101]
HACD1	Protein Tyrosine Phosphatase-Like (PTPLA) now referred to as 3-Hydroxyacyl-CoA Dehydratase 1	Genomic methods have indicated that HACD1 plays a role in AD	[141]
HMGA1	High Mobility Group AT-Hook 1	Induces aberrant exon skipping of Presenilin-2 (PS2) RNA, in sporadic Alzheimer’s disease	[93]
HSP90	Human Hsp90	May form a toxic complex with Tau in AD	[142]
ILK	Integrin-linked Kinase	Expression rescued hippocampal neurogenesis and memory deficits in an AD animal model	[143]
KIF12	Brain specific protein Kinesin	Play a role in cellular transport and secretion,	[89, 114, 144]
KIR222 KIR3DP1	Killer inhibitory receptors	Killer inhibitory receptors similar to KIR222 and KIR3DP1 were associated with Alzheimer’s disease	[102, 103]
L3MBTL2	Genomic variants of Histone Methyl-Lysine Binding Protein 2	Were significantly associated with AD	[105]
MAP3K19	Dual Leucine Zipper-Bearing Kinases similar to Mitogen-Activated Protein Kinase Kinase Kinases (MAPKKK)	May play a role in Neuronal Development and Stress Management	[145]
METTL17	Methyltransferases	The inhibition of methyltransferases rescued synaptic and cognitive functions for Alzheimer’s disease	[106]
NET1	RHOGEF Neuroepithelial cell-transforming 1	Is involved in cell proliferation in neurological development	[107]
NEUROG2	Basic helix-loop-helix (bHLH) transcription factor Neurogenin 2	Plays a role in the development of Neurons via the Wnt/β-Catenin pathway	[108]
NGF	Nerve Growth Factor	Nerve growth factor governs the development of neurons	[4]
NOX1	NADPH Oxidase	May play a role in neurodegenerative disorders	[146]
NPR2	Natriuretic Peptide Receptor 2	Internalization of amyloid-β sensitive to natriuretic peptides	[109, 110]
NT5C3A	5'-Nucleotidase	May be expressed in the early stages of Alzheimer’s disease	[147]
NUP210	Nucleoporin 210	The gene is active specifically in the prefrontal cortex neurons	[148]
NXNL1	Rod-Derived Cone Viability Factor Nucleoredoxin Like 1	Loss of optical sensory nerve cells in retinitis pigmenstosa	[97]
NXNL1	Nucleoredoxin Like 1	TAU phosphorylation is increased in NXNL1 (−/−) mice	[95]
OGFOD3	2-Oxoglutarate-dependent Dioxygenases	Sense energy metabolism, oxygen and iron homeostasis that might have a role in aging	[149]
OR8B12	Olfactory receptor 8B	Known to be expressed in the brain and is a target of autoimmune response in AD	[83]
OSBPL1A	Like Oxysterol-binding protein-1 (OSBP1)	Modulates processing and trafficking of the amyloid precursor protein	[150]
PCGF2	Polycomb Group Ring Finger 2	May be involved in complexes that participate in amyloid signalling in neurodegenerative disorders	[151]
PDCD5	Programmed Cell Death 5	May play a role in programmed cell death observed in neurodegenerative disorders	[111]
PDE4C	Phosphodiesterase 4C	Inhibition of phosphodiesterase investigated for the treatment of AD	[152]
PDZD9	PDZ Domain proteins	Interact with amyloid precursor protein	[153]
PHACTR4	Phosphatase and actin regulator 4	Regulates Actin Dynamics and Cofilin-Actin Rods in AD	[154]
PRKCD	Protein Kinase C Delta	Is associated with a dysregulated Fc Gamma Receptor-mediated phagocytosis pathway in AD	[104]
PRKN	Parkinson Protein 2, E3 Ubiquitin Protein Ligase formerly PARK2	Deubiquitinating Enzymes Regulate PRKN -mediated Mitophagy neurodegenerative disorders	[120]
RAD52	RAD52 Homolog, DNA Repair Protein	Defects in RAD52 Homolog, DNA Repair Protein (RAD52) may contribute to neurodegeneration in AD	[155]
SGK3	Serum/Glucocorticoid Regulated Kinase Family Member 3	May reflect the role of glucocorticoid receptors in AD	[156]
SLC24A2	Solute Carrier Family 24 (Sodium/Potassium/Calcium Exchanger), Member 2	Genomic evidence has shown associations between mutations in SLC24A4 in AD	[157]
SLC30A1	Solute Carrier Family 30 Member 1 (SLC30A1)	Alterations in Zinc Transporter protein observed in the brain of subjects with AD	[158]
SMPD3	Sphingomyelin Phosphodiesterase 3	Deficiency causes progressive cognitive impairment	[91]
SORBS3	Genomic mutations in Sorbin and SH3 Domain Containing 3	Were associated with Alzheimer’s disease	[112]
SPEG UBE2L3	Striated Muscle Enriched Protein Kinase & Ubiquitin Conjugating Enzyme E2 G1	SPEG and UBE2L3 were differentially methylated genes associated with cognitive impairment	[40, 41]
SYTL4	Synaptotagmin Like 4	Exocytosis or secretion from neurological synapses	[92]
TAGLN2	Transgelin 2	Quantitative protein profiling of Hippocampus showed that Transgelin 2 (TAGLN2) expression increased during human aging [159]	[159]
TBATA	Thymus, Brain And Testes Associated	Role in neurite outgrowth increased in the circulation in AD	[114]
TNF	Tumor Necrosis Factor	TNF degradation products in plasma were observed herein but a recent review concludes there is no increase in circulating TNF- α in Alzheimer’s disease [115]	[115]
TNFRSF14	TNF Receptor Superfamily Member 14	Genetic Deletion of TNF Receptor Superfamily member II, enhanced AD Pathology in an Mouse Model	[116]
TNIK	TRAF2 and NCK Interacting Kinase	Observed in inclusion body-like structures in cognitively impaired and genetic association study indicated that TNIK gene variants had a significant association with Alzheimer-type dementia risk	[118]
TPH1	Tryptophan Hydroxylase 1	Was mis-regulated in the human hippocampus in AD	[160]
TRAF4	TNF Receptor Associated Factor 4	Expression of TNF Receptor Associated Factor similar to TRAF4 was observed in Mouse Models of Down's Syndrome and Alzheimer’s disease	[117]
TRIML2	Polymorphisms in Tripartite Motif Family-Like 2	TRIM14 associated with Alzheimer’s disease Risk	[119]
VTI1B	Vesicle Transport Through Interaction With T-SNAREs 1B	Cell death occurs upon loss of t-SNAREs that may interact with VTI1B	[121, 122]
WDR82	WD repeat-containing protein 82	Neurodegeneration was associated with mutations in the WD repeat domain 45 (WDR45)	[161]
ZC3H12A	Zinc Finger CCCH-Type Containing 12A Endoribonuclease	Genetic variation in imprinted genes such as ZC3H12A is associated with the risk of late-onset Alzheimer’s disease	[162]
ZNF671	Zinc Finger Proteins nucleic acid binding and transcription	Exome Sequencing identified Alzheimer's-Associated Variants in zinc finger domain protein ZNF655	[163, 164]

### Processing of DISC1 in AD versus matched controls

**Fig. 6:**
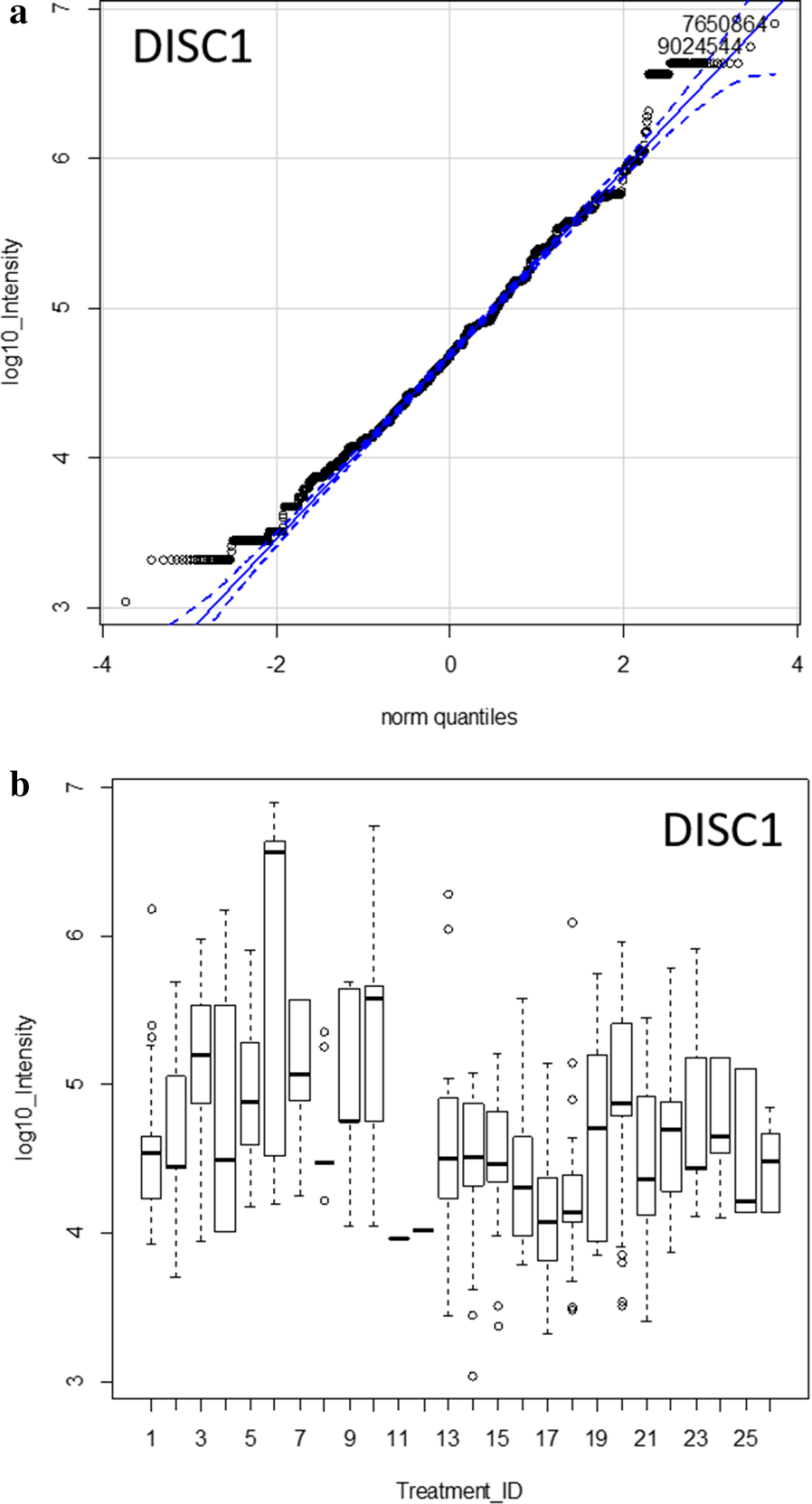
The quantile and box plot of all DISC1 peptides compared over disease treatments. Panels:** a** quantile plot showing the Gaussian intensity distribution; **b** the quantile box plot showing the intensity distribution of each treatment. Treatment ID numbers: 1, Alzheimer normal; 2, Alzheimer’s normal control STYP; 3, Alzheimer’s dementia; 4, Alzheimer’s dementia STYP; 5, Cancer breast; 6, Cancer breast STYP; 7, Cancer control; 8, Cancer control STYP; 9, Cancer ovarian; 10, Cancer ovarian STYP; 11, Ice Cold; 12, Ice Cold STYP; 13, Heart attack Arterial; 14 Heart attack Arterial STYP; 15, Heart attack normal control, 16, Heart attack normal Control STYP; 17, Heart attack; 18, Heart attack STYP; 19, Multiple Sclerosis normal control; 20, Multiple Sclerosis normal control STYP; 21, Multiple sclerosis; 22, Multiple sclerosis STYP, 23 Sepsis; 24, Sepsis STYP; 25, Sepsis normal control; 26, Sepsis normal control STYP. There was significant effects of treatments and peptides by one way and two-way ANOVA

**Fig. 7:**
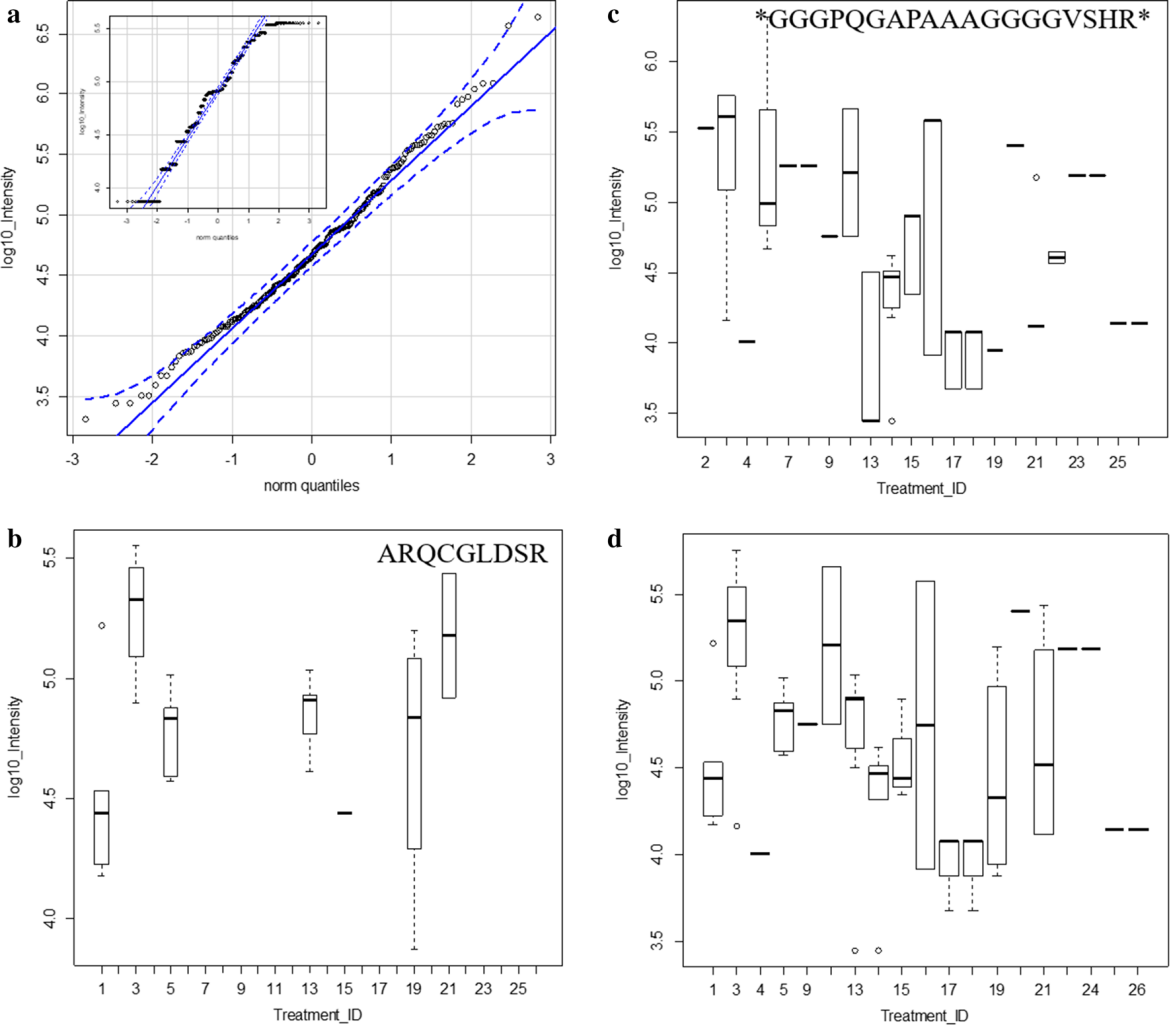
The intensity distributions of the peptides from the conserved N terminus of DISC1 across disease and controls treatments. Panels: **a** The quantile plot of all DISC1 peptide intensity from within the sequence MPGGGPQGAPAAAGGGGVSHRAGSRDCLPPAACFR (inset, the quantile plot of the selected DISC1 peptides ARQCGLDS; **b** the quantile box plot of the DISC1 peptide ARQCGLDS; **c** the quantile box plot of the DISC1 peptides within the sequence MPGGGPQGAPAAAGGGGVSHRAGSRDCLPPAACFR; **d** the quantile box plot of the DISC1 peptides from ARQCGLDS and within the sequence MPGGGPQGAPAAAGGGGVSHRAGSRDCLPPAACFR. Treatment ID numbers: 1, Alzheimer normal; 2, Alzheimer’s normal control STYP; 3, Alzheimer’s dementia; 4, Alzheimer’s dementia STYP; 5, Cancer breast; 6, Cancer breast STYP; 7, Cancer control; 8, Cancer control STYP; 9, Cancer ovarian; 10, Cancer ovarian STYP; 11, Ice Cold; 12, Ice Cold STYP; 13, Heart attack Arterial; 14 Heart attack Arterial STYP; 15, Heart attack normal control, 16, Heart attack normal Control STYP; 17, Heart attack; 18, Heart attack STYP; 19, Multiple Sclerosis normal control; 20, Multiple Sclerosis normal control STYP; 21, Multiple sclerosis; 22, Multiple sclerosis STYP, 23 Sepsis; 24, Sepsis STYP; 25, Sepsis normal control; 26, Sepsis normal control STYP. There was significant effects of treatments and peptides by two-way ANOVA

The DISC1 protein that best fit the MS/MS spectra observed from human plasma was accession AAH07022.1. The average peptide intensity per gene symbol of DISC1 was higher in AD compared to AD control (Fig. 6). The processing of DISC1 included the cleavage of the terminal peptide MPGGGPQGAPAAAGGGGVSHR* and ARQCGLDSR from two hydrophilic points of DISC1 on the conserved amino terminal domain of the protein (Fig. 7) that was apparent in AD patients compared to all other diseases and controls. Thus, there was disease associated variation in the processing of DISC1 in AD versus AD controls or other diseases and normal (Table 4).

**Table 4:** The analysis of log_10_ peptide intensity DISC1 protein by ANOVA from all DISC1 (NP_001158009) peptides and from the selected peptides ARQCGLDS & MPGGGPQ* for means comparison

Treatment_ID	Log10 Intensity	se	N	Log10 intensity	se	N
	All Peptides			ARQCGLDS & MPGGGPQ*		
1	4.54	0.12	13	4.52	0.19	5
2	4.64	0.16	10	NA	NA	NA
3	5.14	0.09	28	5.27	0.10	16
4	4.52	0.21	7	4.01	NA	1
5	5.03	0.14	8	4.78	0.09	5
6	5.48	0.65	4	NA	NA	NA
7	5.07	0.18	4	NA	NA	NA
8	4.47	NA	1	NA	NA	NA
9	4.97	0.20	7	4.75	0.00	2
10	5.47	0.13	10	5.21	0.26	4
13	4.47	0.13	20	4.67	0.16	9
14	4.58	0.15	10	4.27	0.21	5
15	4.43	0.10	14	4.56	0.17	3
16	4.42	0.19	8	4.75	0.83	2
17	4.10	0.16	10	3.94	0.13	3
18	4.65	0.25	11	3.94	0.13	3
19	4.57	0.18	10	4.44	0.24	6
20	4.81	0.28	6	5.40	0.00	2
21	4.54	0.17	10	4.65	0.32	4
22	4.62	0.14	8	NA	NA	NA
23	4.79	0.21	8	5.18	0.00	2
24	4.73	0.16	6	5.18	0.00	2
25	4.47	0.22	4	4.14	NA	1
26	4.41	0.27	2	4.14	NA	1

Treatment ID numbers: 1, Alzheimer normal; 2, Alzheimer’s normal control STYP; 3, Alzheimer’s dementia; 4, Alzheimer’s dementia STYP; 5, Cancer breast; 6, Cancer breast STYP; 7, Cancer control; 8, Cancer control STYP; 9, Cancer ovarian; 10, Cancer ovarian STYP; 11, Ice Cold; 12, Ice Cold STYP; 13, Heart attack Arterial; 14 Heart attack Arterial STYP; 15, Heart attack normal control, 16, Heart attack normal Control STYP; 17, Heart attack; 18, Heart attack STYP; 19, Multiple Sclerosis normal control; 20, Multiple sclerosis normal control STYP; 21, Multiple sclerosis; 22, Multiple Sclerosis STYP, 23 Sepsis; 24, Sepsis STYP; 25, Sepsis normal control; 26, Sepsis normal control STYP

### DISC1 domains

**Fig. 8:**
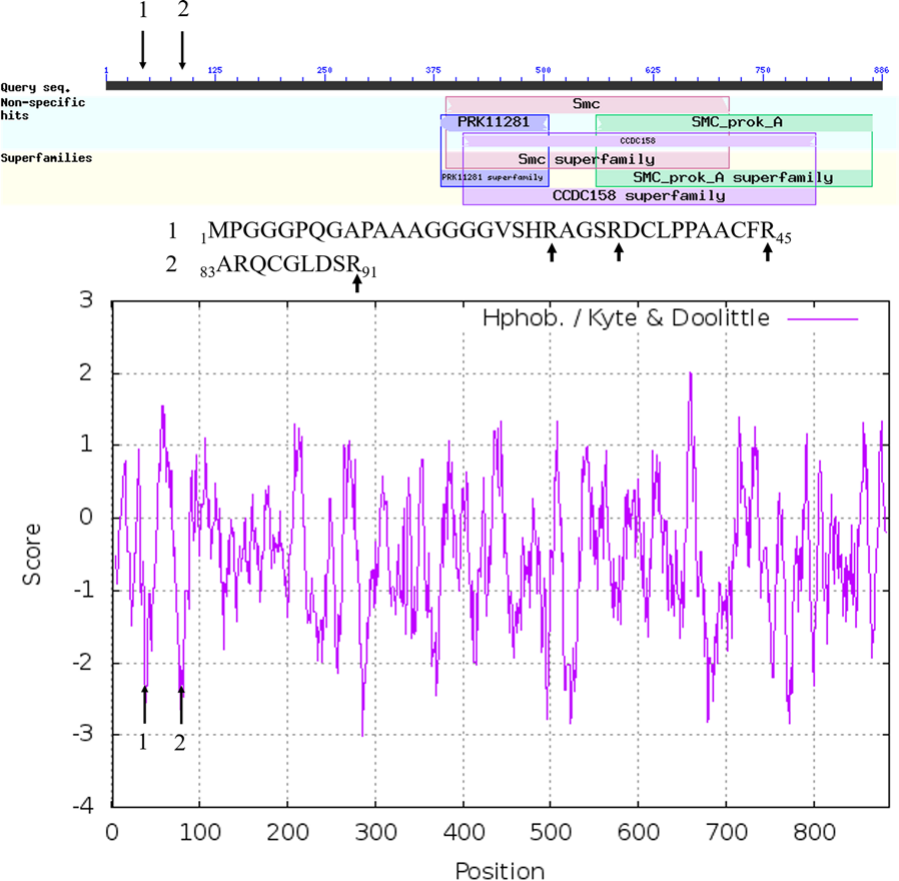
The primary structure and hydrophobicity plot of Disrupted in Schizophrenia 1 protein isoform a [Homo sapiens] DISC1 NP_001158009.1. The long arrow shows the cleavage site of the tryptic peptides sequences: 1, _1_MPGGGPQGAPAAAGGGGVSHRAGSRDCLPPAACFR_45_; and 2, _83_ARQCGLDSR_91_ from the conserved and unique C-terminal domain of DISC1 that is conserved within humans and across mammals in sequences available to date. The short arrows show the location of the tryptic cleavage sites observed

The cleavage of specific peptides from the conserved N-terminal domain of DISC1 was most frequently observed in AD compared to all other treatments and showed greater intensity compared to other treatments. Thus, the processing of DISC1 in AD patients apparently varied compared to all other diseases and controls. The function and mechanisms of DISC1 in Alzheimer dementia are not clear [44–47, 87]. There may be some hints about the function of DISC1 by the examination of its domain architecture (Fig. 8). DISC 1 shows some significant similarity with the SMC and SMC_prok_A domain families associated with chromosome segregation, has homology with a mechanosensitive channel MscK, and is the lone member of the coiled-coil CCDC158 superfamily (Table 5).

**Table 5:** The conserved domains of DISC1 (NP_001158009) from NCBI BLAST analysis and Domain Architecture retrieval tool (DART)

Name	Accession	Description	Interval	E-value
Smc	COG1196	Chromosome segregation ATPase, Cell cycle control, cell division	388–711	1.25E−05
PRK11281	PRK11281	Mechanosensitive channel MscK	382–506	0.000816
SMC_prok_A	TIGR02169	Chromosome segregation protein SMC, primarily archaeal type	559–874	0.00211
CCDC158	pfam15921	Coiled-coil domain-containing protein 158, CCDC158	408–810	0.00274

## Discussion

Comparison of AD plasma to matched controls, alongside other disease and normals under identical conditions is a simple and direct strategy to discover variation in peptides or proteins specific to AD plasma. The aim and objective of this study was proof of concept towards a method to compare the endogenous tryptic peptides of AD to those from AD control and other diseases or normals by random and independent sampling with a set of robust and sensitive linear quadrupole ion traps where the results were collected in an SQL Server for analysis with the R statistical system. Although random and independent sampling of peptides from step-wise fractionation of plasma followed by LC–ESI–MS/MS is a time and manual labor intensive approach, it is sensitive, direct, and rests on few assumptions [49, 88]. High signal-to-noise ratio of blood peptides is dependent on sample preparation to break the sample into many sub-fractions to relieve competition and suppression of ionization and thus achieve sensitivity [55, 56, 68] but then requires large computing power to re-assemble the sub-fractions, back into individual patient samples within treatments [55, 60, 88]. The approach shows great sensitivity and flexibility but relies on the fit of MS/MS spectra by X!TANDEM and SEQUEST [57, 58] to assign peptide identity and statistical analysis of precursor ion counts and intensity by Chi Square and ANOVA and so is computationally intensive [60, 61]. The careful study of plasma degradation over time, and under various storage and preservation conditions, seems to rule out pre-clinical variation as the most important source of variation between AD versus AD control or other disease and control treatments [49, 65, 82]. Proteins expressed in AD within the brain may be identified in the blood [1]. Proteins linked to the mechanisms of AD pathology from nuclear factors of transcription, and exon processing, proteins for transport or secretion and signalling proteins associated with regulating cell survival and cell cycle as well as innate immune response and the cell-death, i.e. apoptotic, necrotic, necroptic and/or phagocytic pathways were observed with Alzheimer’s dementia [89].

### STRING analysis

Analysis of the proteins frequently observed from endogenous peptides in AD versus control clinical sample sets using Chi Square analysis was a direct means to look for factors specific to AD that might play a role in the mechanism of AD pathology for analysis by the STRING algorithm [86]. The large number of edge connections between the gene symbols specific to AD indicated the proteins observed were not a random assemblage of factors but show biological and protein- interactions consistent with bone fide biological variation between the AD versus AD control treatments. The observed proteins associated with alternate splicing, exocytosis of luminal proteins, and the regulation of the cell survival, mitochondrial functions or metabolism including the metabolism of ceramides [90] and apoptosis were consistent with the known mechanisms of AD pathology described below.

### AD versus AD control alongside other diseases and control by ANOVA

Proteins that showed increased observation frequency in AD versus AD control by Chi Square (χ2) were also then analyzed across all disease and control treatments by box plots, quantile plots and ANOVA. The complete analysis of mean precursor intensity [62–64] may require two way ANOVA [61]. Examining the gene symbol intensity across all twelve disease and control clinical sample sets by box plots and two-way ANOVA was a direct means to compare across all other diseases and controls to look for proteins specific to AD. Many of the proteins that show increased observation in AD independently showed greater log_10_ intensity values that was consistent with true-positive variation between AD and AD control. Analysis of peptides across all treatments will be required to extract all of the information from such as large dataset and will require large and automated computations.

### Agreement with previous genetic and biochemical experiments

There was excellent agreement between the proteins observed in the plasma of AD patients and the previous genetic and biochemical evidence for the mechanisms of Alzheimer’s dementia at all cellular levels from transporters, receptors, cellular metabolic and signalling enzymes, transcription factors and DNA/RNA binding factors in the results. For example, Sphingomyelin Phosphodiesterase 3 (SMPD3) deficiency causes progressive cognitive impairment [91]. Synaptotagmin Like 4 (SYTL4) functions in exocytosis or secretion from neurological synapses and so synaptotagmins may be considered as possible AD biomarkers [92]. Many proteins showed known connections to the mechanisms of AD pathology such as amyloid or Tau pathways including secretases and presenilin. For example HMGA1 induces aberrant exon skipping of Presenilin-2 (PS2) RNA, in sporadic Alzheimer’s disease [93]. A chymotrypsin like activity results in a carboxyl-terminal-truncated Apolipoprotein E4 that causes Alzheimer’s disease-Like Neurodegeneration in mice [94]. TAU phosphorylation is increased in Nucleoredoxin Like 1 (NXNL1) (−/−) mice [95]. In contrast, the role of SMIM19 and many other proteins remain entirely obscure with respect to AD.

However, a subset of the AD-specific proteins that were directly related to the cell proliferation, death/ survival and cell clearance pathways will be emphasized here. Nerve growth factor governs the growth development of neurons [4]. The ataxia-telangiectasia mutated (ATM) protein kinase is associated with neurodegeneration and is a master regulator of the DNA damage response that may be activated by Nibrin, i.e. NBS1(NBN) [96]. The Rod-Derived Cone Viability Factor Nucleoredoxin Like 1 (NXNL1) that governs the loss of optical sensory nerve cells in retinitis pigmenstosa has a direct connection to the regulation of cell death [97]. The cell death-associated protein kinase family (DAPK) may be therapeutic targets [98]. Variants in the splicing regulatory elements of EXOC3L4 were associated with Alzheimer’s disease [99]. GRID2 is important for the function of the NMDA receptor that plays a key role in synaptogenesis, synaptic plasticity, and motor coordination and that is a therapeutic target in AD [100]. Glutaredoxin And Cysteine Rich Domain Containing 1 (GRXCR1) that may function in cell survival was observed in the plasma of AD patients and Glutaredoxin was released to the cerebrospinal fluid in the early stages of AD [101]. Killer inhibitory receptors similar to KIR222 and KIR3DP1 that may function in cell clearance were associated with Alzheimer’s disease [102, 103]. Protein Kinase C Delta (PRKCD) is associated with a dysregulated Fc Gamma Receptor-mediated phagocytosis pathway in Alzheimer’s disease [104]. Genomic variants of Histone Methyl-Lysine Binding Protein 2 (L3MBTL2) that may function in the survival of motor neurons were significantly associated with AD [105]. The inhibition of methyltransferases that are functionally or structurally related to METTL17 rescued synaptic and cognitive functions for Alzheimer’s disease [106]. The RHOGEF Neuroepithelial cell-transforming 1 (NET1) is involved in cell proliferation in neurological development [107]. The basic helix-loop-helix (bHLH) transcription factor Neurogenin 2 (NEUROG2) plays a role in the development of Neurons via the Wnt/β-Catenin pathway [108]. The observed changes in Natriuretic Peptide Receptor 2 (NPR2) may reflect the internalization of amyloid-β Peptide in brain capillary endothelial cells [109, 110]. Molecules like Programmed Cell Death 5 (PDCD5) may play a role in programmed cell death observed in neurodegenerative disorders [111]. Genomic mutations in Sorbin and SH3 Domain Containing 3 (SORBS3) that may regulate cell proliferation were associated with Alzheimer’s disease [112, 113]. Thymus, Brain And Testes Associated (TBATA) that plays a role in neurite outgrowth increased in the circulation in AD [114]. Greater levels of TNF degradation products in plasma were observed herein but a recent review concludes there is no increase in circulating TNF- α in Alzheimer’s disease [115] perhaps indicating a role for turnover in the regulation of TNF levels. Genetic Deletion of TNF Receptor Superfamily member II, that is functionally similar to TNFRSF14, enhanced the Alzheimer-like Pathology in an APP Transgenic Mouse Model [116]. Expression of TNF Receptor Associated Factor similar to TRAF4 was observed in mouse Models of Down's Syndrome and Alzheimer’s disease [117]. TRAF2 and NCK Interacting Kinase (TNIK) was observed in inclusion body-like structures in cognitively impaired and genetic association study indicated that TNIK gene variants had a significant association with Alzheimer-type dementia risk [118]. The tripartite motif (TRIM) proteins, characterized by the RING, B-Box and coiled-coil (RBCC) domains at the N-terminus, interact with p53 to regulate cell proliferation/death and polymorphisms in Tripartite Motif Family-Like 2 (TRIML2) that is structurally or functionally similar to TRIM14 were associated with Alzheimer’s disease Risk [119]. Deubiquitinating Enzymes Regulate PARK2-mediated Mitophagy is implicated in many neurodegenerative disorders like Alzheimer’s disease [120]. Presenilin controls kinesin-1 and dynein function during APP-vesicle Transport in vivo and cell death occurs upon loss of t-SNAREs [121, 122] that may interact with protein such as Vesicle Transport through Interaction with T-SNAREs 1B (VTI1B).

### Structure and functions of DISC1

The DISC1 gene that is disrupted in schizophrenia encodes coiled-coil domain protein conserved in bacteria and eukaryotes that functions in chromosome segregation and structural maintenance of chromosomes with homology to SMC and SMC_prok_A domains that may localize to the centrosome and punctate cytoplasmic foci and is classified as a scaffold protein due to its established interactions with several other proteins including AA598-854 activating transcription factors 4 and 5 (ATF4 and ATF5) and Microtubule Associated Protein 1A (MAP1A) [123], Platelet Activating Factor Acetylhydrolase 1b Regulatory Subunit 1 (PAFAH1B1), Pericentrin (PCNT) [124, 125] and Interaction with NudE Neurodevelopment Protein 1 Like 1 (NDEL1) [126, 127]. DISC1 is involved in neurogenesis that is regulated by WNT signalling leading to neural progenitor proliferation by modulating GSK3B activity and CTNNB1 abundance [128, 129] and inhibits AKT-mTOR upon interaction with CCDC88A [130, 131]. The apparent functions of DISC1 in regulating genomic organization and gene expression that may influence neuronal development are consistent with the variation in DISC1 peptides observed in Alzheimer’s dementia. Thus, it might be possible to detect and resolve AD patients from the background population of AD controls by monitoring the levels and/or processing of DISC1 in EDTA plasma.

## Conclusion

It was possible to discover peptides and/or proteins that showed variation specific to AD versus other diseases, or normal plasma samples, from many institutions using disposable sample preparation, common bench-top instrumentation, and generic computation. The LC–ESI–MS/MS of plasma endogenous tryptic peptides identified many blood proteins and/or peptides in AD versus AD control that were previously associated with the innate immune response. The observation frequency and intensity of proteins specific to AD agreed with STRING analysis of known interactions and the previous genetic and biochemical evidence that the peptides and proteins specific to AD showed statistical and biological significance with respect to marking the mechanisms of the disease process including aberrant RNA metabolism. Cleavage of the DISC1 protein to release peptides from the COOH terminal and elsewhere was more frequent in AD compared to all other diseases and controls. DISC1 peptides discovered by random and independent sampling of test samples might be confirmed by automatic targeted LC–ESI–MS/MS [49, 65, 82] from a larger cohort of independent samples.

## Supplementary Information

**Additional file 1: Table S1.** The list of protein Gene Symbols with Chi Square (χ2) values greater than nine (9) at 1 degree of freedom.

**Additional file 2: Table S2.** The STRING Keyword analysis with Chi Square (χ2) values greater than nine (9) at 1 degree of freedom.

**Additional file 3: Table S3.** The STRING Component analysis with Chi Square (χ2) values greater than nine (9) at 1 degree of freedom.

**Additional file 4: Table S4.** The STRING Function analysis with Chi Square (χ2) values greater than nine (9) at 1 degree of freedom.

**Additional file 5: Table S5.** The STRING Process analysis with Chi Square (χ2) values greater than nine (9) at 1 degree of freedom.

**Additional file 6: Table S6.** The replication of the LC–ESI–MS/MS experiments.

## Data Availability

The raw data is provided in companion publication and the supplemental data.

## Abbreviations

AD: Alzheimer’s Disease
TRYP: Fully tryptic
STYP: S, T or Y tryptic phosphopeptide and/or fully tryptic
